# On the Temperature of the Earth and Sea, in Reference to the Theory of Central Heat: A Lecture Delivered at the Royal Institution

**Published:** 1846-07

**Authors:** 


Art. VI.—*On the Temperature of the Earth and Sea, in reference to the Theory of Central Heat: a Lecture delivered at the Royal Institution.* By Alfred S. Taylor, f.r.s., &c.—*London,* 1846. 8vo, pp. 32.

This is an excellent Lecture on a very interesting point of scientific inquiry. It contains all the knowledge we possess on the subject of which it treats, and this knowledge disposed clearly and in an agreeable form. We think there are few readers whom it will not interest, and not many whom it will not instruct.

